# Safety and effectiveness of LEO stents for dual stent-assisted embolization combined with IA and IV intra-procedural infusion of tirofiban in the treatment of wide-necked intracranial bifurcation aneurysms

**DOI:** 10.3389/fneur.2024.1393310

**Published:** 2024-07-10

**Authors:** Kaishan Wang, Zhaopan Lai, Zenan Zhao, Jun Tang, Cheng Yang, Biao Yang, Gang Zhu, Hongping Miao

**Affiliations:** ^1^Department of Neurosurgery, First Affiliated Hospital of Army Medical University, Chongqing, China; ^2^Department of Neurosurgery, Chongqing Western Hospital, Chongqing, China; ^3^Department of Neurosurgery, Chongqing Medical University Pediatric College, Children's Hospital of Chongqing Medical University, Chongqing, China; ^4^Department of Neurosurgery, The Affiliated Dazu's Hospital of Chongqing Medical University, Chongqing, China

**Keywords:** dual stents, wide-necked intracranial bifurcation aneurysm, antiplatelet protocol, tirofiban, efficacy, safety

## Abstract

**Objective:**

To evaluate the safety and efficacy of employing LEO stents in dual stent-assisted embolization (DSAE) for wide-necked intracranial bifurcation aneurysms, and to assess the effectiveness of combined IA and IV intra-procedural infusion of tirofiban in mitigating perioperative complications.

**Methods:**

Clinical data and follow-up images from 562 patients with wide-necked intracranial bifurcation aneurysms treated at First Affiliated Hospital of Army Medical University from 2018–2022 were collected. Among them, 65 received DSAE with LEO stents. The study observed treatment success rates, procedure-related complications, perioperative thromboembolic events (TEs) and hemorrhagic events (HEs), immediate postoperative modified Raymond-Roy classification (mRR), and follow-up imaging. Glasgow Outcome Scale (GOS) at discharge and clinical follow-ups were recorded.

**Results:**

The study enrolled 65 patients (mean age: 56.77 ± 10.07) with wide-necked intracranial bifurcation aneurysms. Among them, 58 had unruptured aneurysms, 7 ruptured (Hunt-Hess II-III). All aneurysms were successfully embolized without significant stent or bleeding complications. Only one case had intraoprative thrombosis; two postoperative ischemic incidents occurred within three days, no severe bleeding events. Immediate imaging showed modified Raymond-Roy classification: mRRC I (92.3%), mRRC II (4.6%), mRRC III b (3.1%). A total of 43 patients were followed up postoperatively with DSA. Among them, 41 patients exhibited mRRC I, while 2 patients exhibited mRRC II. No aneurysm was recanalized. Discharge GOS: GOS 5–60, GOS 4–1, GOS 3–4. One patient, GOS 1, died from lung cancer; others improved.

**Conclusion:**

The utilization of LEO stents for dual stent-assisted embolization of wide-necked intracranial bifurcation aneurysms demonstrated remarkable success and safety, yielding favorable postoperative outcomes and no instances of aneurysm recurrence. The concomitant administration of perioperative antiplatelet medications alongside IA and IV intra-procedural infusion of tirofiban effectively attenuated thromboembolic events (TEs) without concomitant elevations in bleeding risks.

## 1 Introduction

The use of stent-assisted coiling (SAC) is a well-established and widely accepted method for the endovascular treatment of wide-neck intracranial aneurysms (1). Conventional stent-assisted embolization of intracranial wide-necked aneurysms is safe and effective but is not as effective for wide-necked intracranial bifurcation aneurysms. Although several treatment methods are available, including the balloon-assisted technique, multicatheter technique, and lantern technique (2), limitations such as a low rate of complete occlusion of the aneurysm and high susceptibility of the spring coil to dislodgement pose challenges in treatment, especially when protecting branch vessels. The dual stent-assisted embolization technique proposed by Chow et al. maintains the fluency of the affected branch by implanting a stent in the branch vessel and creating a new bifurcation site at the bifurcation aneurysm neck, which can stabilize the coil inside the aneurysm capsule and prevent the coil from herniated into the branch vessel (3–5). In the early stages of dual stent-assisted embolization, carved stents were commonly used. Despite their high success rate, their design made them prone to fracturing at sharp vascular bends. Their low metal coverage also led to inadequate repair of the aneurysm neck, increasing the likelihood of recurrence. Braided stents such as LEO stents have better vascular compliance, and releasing operations can better facilitate stent opening and adherence to walls at turning points (6). The design of the LEO sliding unit grids can enable stents to open fully at crossing sites intraoperatively; additionally, the stent grid is smaller and has greater metal coverage, which can more effectively promote the growth of the vascular endothelium and increase vascular repair ability (6–9). This technique has been used to treat complex wide-necked intracranial bifurcation aneurysms. However, after the implantation of two braided stents in the aneurysm-carrying artery, the blood flow in the aneurysm capsule decreases, which facilitates thrombosis in the aneurysm capsule but increases the risk of perioperative TEs. Previously, perioperative thromboembolic events (TEs) were prevented through oral dual antiplatelet therapy, yet the incidence of perioperative TEs remained as high as 11.5% (5, 10). In our center, perioperative TEs were prevented by oral dual antiplatelet therapy combined with IA and IV intra procedural infusion of tirofiban. In this retrospective study, we analyzed the clinical outcomes of 65 cases involving intracranial wide-necked aneurysms treated with dual stent-assisted embolization utilizing LEO stents at our institution. We assessed the efficacy of embolization treatment for these aneurysms and evaluated the effectiveness of tirofiban administration, in conjunction with IA and IV intra-procedural infusion, in preventing perioperative TEs.

## 2 Materials and methods

### 2.1 Clinical data and treatment methods

**Figure 1:**
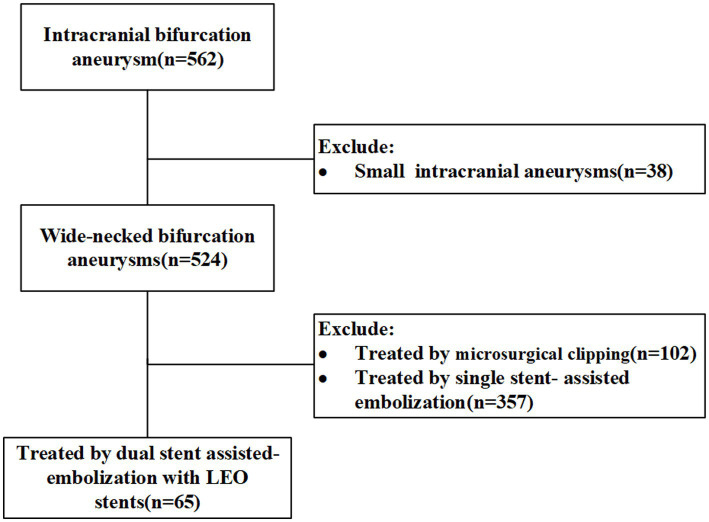
Flowchart for patient selection.

The clinical data of 562 patients with wide-necked intracranial bifurcation aneurysms who were admitted to the Neurosurgery Department of the First Affiliated Hospital of the Army Medical University were analyzed retrospectively from 2018 to 2023. Among them, 65 patients were treated with dual stent-assisted embolization using LEO stents. In this study, wide-necked intracranial aneurysms were defined as those with aneurysm necks ≥4 mm or dome-to-neck ratios ≤2. The inclusion criteria for patients were as follows: (1) dual-stent assisted embolization of wide-necked intracranial bifurcation aneurysms using LEO stents and (2) the age ranged from 18 to 80 years. The exclusion criterion was a microaneurysm in the BIA. Intracranial bifurcation aneurysm is defined as an aneurysm situated at the bifurcation of major blood vessels, including the terminus of the basilar artery, the bifurcation of the middle cerebral artery (MCA), the anterior communicating artery (ACoMA), the terminus of the internal carotid artery (ICA), or other smaller arteries. The flowchart is shown in Figure 1. The study was approved by the Ethics Committee of the First Affiliated Hospital of Army Medical University. We certify that the study was performed in accord with the 1964 declaration of HELSINKI and later amendments. The demographic data, aneurysm location and size, previous medical history, stent shaping technique, stent placement success rate, intraoperative complications, degree of aneurysm occlusion, postoperative complications and management were retrieved from the patients’ medical records.

### 2.2 Perioperative antiplatelet protocol

In the present study, 58 patients with unruptured intracranial aneurysms received oral dual antiplatelet drug therapy (baiaspirin: 100 mg, clopidogrel bisulfate tablets: 75 mg of clopidogrel bisulfate tablets) for 3–5 days before surgery. During the procedure, intravenously administer tirofiban through a catheter at a dose of 10 μg/kg over 3 min, while simultaneously infusing tirofiban intravenously at a rate of 0.1 μg/kg/min. This infusion lasts for 24 to 48 h post-surgery. In the final 6 h before discontinuation, bridge with 100 mg aspirin and 75 mg clopidogrel orally; in seven patients with ruptured aneurysms, no oral antiplatelet drugs were administered before surgery, and the antiplatelet drugs used during and after surgery were the same as those used for unruptured aneurysms; all patients received 75 mg of clopidogrel bisulfate tablets for 6 months and 100 mg of aspirin for 6 months after surgery (7). Platelet function assays were not routinely used or followed because of the restrict China’s Basic Medical Insurance System.

### 2.3 Definition of perioperative TEs and HEs

Perioperative TEs were defined as any angiographic filling defect of the aneurysm-carrying artery or distal arteries during DSA examination and treatment or new ischemic symptoms such as consciousness changes and limb movement changes in the 72-h postoperative period with new low-density shadows or ischemic spots in the vascular distribution area (excluding vasospasm) as clarified by CT or MRI (11). Perioperative HEs were defined as contrast extravasation or herniation of the coil out of the aneurysm capsule during DSA examination and treatment; new intracranial hemorrhage with or without certain clinical symptoms on postoperative head CT/MRI; and other severe bleeding events, such as severe gastrointestinal bleeding, respiratory bleeding, and multiple skin petechiae ecchymoses (12).

### 2.4 Endovascular intervention

All the patients underwent interventional procedures under general anesthesia, which were performed by neurosurgeons specializing in neurosurgery with at least 10 years of surgical experience at our center. Intraoperative heparin was administered intravenously to maintain 2 to 2.5 times the activated clotting time (7). After insertion of the arterial sheath (Terumo), the 6F contrast catheter was passed through the femoral artery to the proximal end of the internal carotid artery or vertebral artery, and the size of the aneurysm was measured via 3D reconstruction of the blood vessels after performing a full cerebral angiogram, analyzing the relationship between the aneurysm-carrying artery and the branch arteries, measuring the diameter of the vessels, and selecting the optimal working angle. The whole procedure was performed under a DSA real-time roadmap, and a 6F or 8F guiding catheter (Microvention, Cordis) was used to guide the aneurysm-carrying artery, and stenting methods were selected according to the characteristics of the aneurysm and the operator’s experience. The selection of the LEO stent is determined solely by measuring the diameter and the distance between the proximal and distal landing zones using 2D and 3D imaging. Based on the diameter of the parent artery and branch vessels, as well as the length of the branch vessels relative to the parent artery, the selection of stent diameter and length is determined. For the initial stent placement, preference is given to placing the stent in larger diameter branch vessels and in vessels with a greater angle relative to the parent artery. Three stenting methods were used: (1) Crossing Y-shaped stent placement: Under micro-guidewire (Stryker; EV3) guidance, navigate a microcatheter (EV3; MicroVention) to the distal straight segment of a branch artery at an acute angle from the aneurysm-carrying artery. Use the microguidewire to guide the spring-coil micro-catheter two-thirds into the aneurysm, deploying an appropriate coil (MicroVention; EV3) to form a basket within the aneurysm sac without detachment. Gradually release the initial longer LEO stent (BALT) through the micro-catheter, immediately administering tirofiban via guiding catheter infusion and simultaneous intravenous delivery, confirming optimal stent expansion via additional angiography. Employ wire-guided manipulation to thread the stent micro-catheter through the first stent’s mesh to the distal end of another branching vessel. In cases of obstruction, stabilize the micro-guidewire, apply controlled pressure to dilate the mesh, and readjust to facilitate successful threading. Partially deploy the second stent to form a Y, infusing tirofiban again through a guiding catheter, proceeding with suitable coil selections to fill the aneurysm and concluding with complete stent deployment. For inadequate opening at the crossing point, utilize the stent’s terminal Y-valve manipulation and overall system manipulation. If necessary, perform post-stent release maneuvers utilizing a curved micro-catheter and wire or balloon dilation techniques for optimal stent expansion. (2) T-shaped stent placement: Guide the stent micro-catheter using a micro-guidewire to one side branch artery, slowly releasing an appropriate LEO stent to ensure full coverage of the aneurysm neck. Confirm optimal stent deployment through angiography, immediately infusing tirofiban via a guiding catheter while maintaining intravenous administration. Using wire-guidance, navigate the stent micro-catheter to the opposite side branch artery, deploy a suitable coil basket using the spring-coil micro-catheter, and semi-release the second stent to create a T configuration with the first stent. Re-infuse tirofiban through the guiding catheter and maintain intravenous administration, continuing with appropriate coil selections to pack the aneurysm sac, culminating in full stent deployment. (3) X-shaped stent placement: Perform femoral artery puncture on the left side, guiding a 6F or 7F guiding catheter under wire guidance to the contralateral internal carotid artery. Use the wire-guided shaped stent micro-catheter to navigate to one side’s anterior cerebral artery A2 segment, deploying an appropriate LEO stent to comprehensively cover one side’s aneurysm neck. Gradually release the stent, confirm coverage adequacy, immediately infusing tirofiban through a guiding catheter while maintaining intravenous administration. Under wire guidance, direct the microcatheter to the distal end of the contralateral anterior cerebral artery A2 segment, guiding the spring-coil microcatheter to two-thirds of the aneurysm, deploying a suitable coil basket, and an appropriate LEO stent partially covering the aneurysm neck. Re-infuse tirofiban through the guiding catheter, maintain intravenous administration, continue with suitable coil selections to fill the aneurysm sac, and finally fully deploy the stents. Following embolization, conduct immediate multi-angle angiography, three-dimensional reconstruction, and post-processing for assessing the extent of aneurysm embolization and stent adherence. Patients who experienced subarachnoid hemorrhage underwent DynaCT imaging as part of routine postoperative assessment, with all patients undergoing bedside CT scans within 12 h following the surgical procedure. Those with subarachnoid hemorrhage received routine head CT and/or MR scans on the 7th day postoperatively and the day preceding discharge to evaluate the extent of cerebral infarction or hemorrhage.

### 2.5 Angiographic and clinical follow-up assessment

Perioperative HEs and TEs during hospitalization were recorded. The clinical outcomes of the patients were assessed by using the Glasgow Outcome Scale (GOS), which was evaluated at the time of discharge from the hospital, and the GOS was followed up at 1 month, 3 months, 6 months, and 2 years after surgery.

The effectiveness of occlusion was assessed by using the cerebral angiographic modified Raymond-Roy (mRR) classification, and the presence of recurrence was first assessed by computed tomography angiography (CTA) or magnetic resonance angiography (MRA) (13). The mRRC was assessed from multiple angles immediately after stent-assisted embolization to determine the effectiveness of occlusion, CTA or MRA was performed for follow-up assessment at 1 and 3 months after the procedure, and DSA cerebral angiography was performed for follow-up at 6 months and 2 years after the procedure.

### 2.6 Statistical analysis

The normality of the distribution of the continuous variables was tested using the skewness-kurtosis test, Kolmogorov–Smirnov test or Shapiro–Wilk test. Continuous variables with a normal distribution are expressed as the mean ± SD. Continuous variables with a nonnormal distribution are expressed as medians and interquartile ranges (IQRs) or ranges. Pearson’s chi-square test or Fisher’s exact test was used to compare ranked variables. A two-tailed *p* value <0.05 was considered to indicate a significant difference.

## 3 Results

### 3.1 Basic features and aneurysm characteristics of the patients

The basic features and aneurysm characteristics of all patients are shown in Table 1. Among them, there were 39 male patients and 26 female patients; their ages ranged from 32 to 77 years old (56.77 ± 10.07); there were 58 cases of unruptured wide-necked intracranial bifurcation aneurysms, and 7 cases of ruptured aneurysms resulting in subarachnoid hemorrhage, with Hunt-Hess grade II – III; there were 43 cases of bifurcation aneurysms of the M1 segment of the middle cerebral artery (MCA), 19 cases of anterior communicating arteries (AcomA), 1 cases of aneurysm at the apex of basilar artery (BA), and 2 cases of aneurysm at the distal A3 segment of the anterior cerebral artery (ACA); the average size of the aneurysm was 4.69 ± 2.85 mm; the average size of the neck of the aneurysm was 4.85 ± 2.43 mm; 25 patients had intracranial aneurysms in other parts of the brain; 37 patients had a history of hypertension, 6 patients had a history of diabetes mellitus, 17 patients had a history of cerebral infarction and so on.

**Table 1:** Summary of demographics, clinical presentation, and aneurysm characteristics.

Demographics	
Age	56.77 ± 10.07
Male/female	39 (60%)/26 (40%)
Risk variables	
Hypertension	37 (56.92%)
Diabetes	6 (9.23%)
Cerebral infarction	17 (26.15%)
Hyperlipidemia	11 (16.92%)
Current smoking	29 (44.16%)
Current drinking	12 (18.46%)
Multiple intracranial aneurysms	25 (38.46%)
Ruptured aneurysm	7 (10.77%)
Unruptured aneurysm	58 (89.23%)
Aneurysm location	
MCA	43 (66.15%)
AcomA	19 (29.23%)
Basilar tip	1 (1.54%)
ACA	2 (3.08%)
Aneurysm size (mm)	4.69 ± 2.85
Neck size (mm)	4.85 ± 2.43
Operation time (h)	3.00 ± 1.11

### 3.2 Immediate angiographic results and perioperative complications

**Figure 2:**
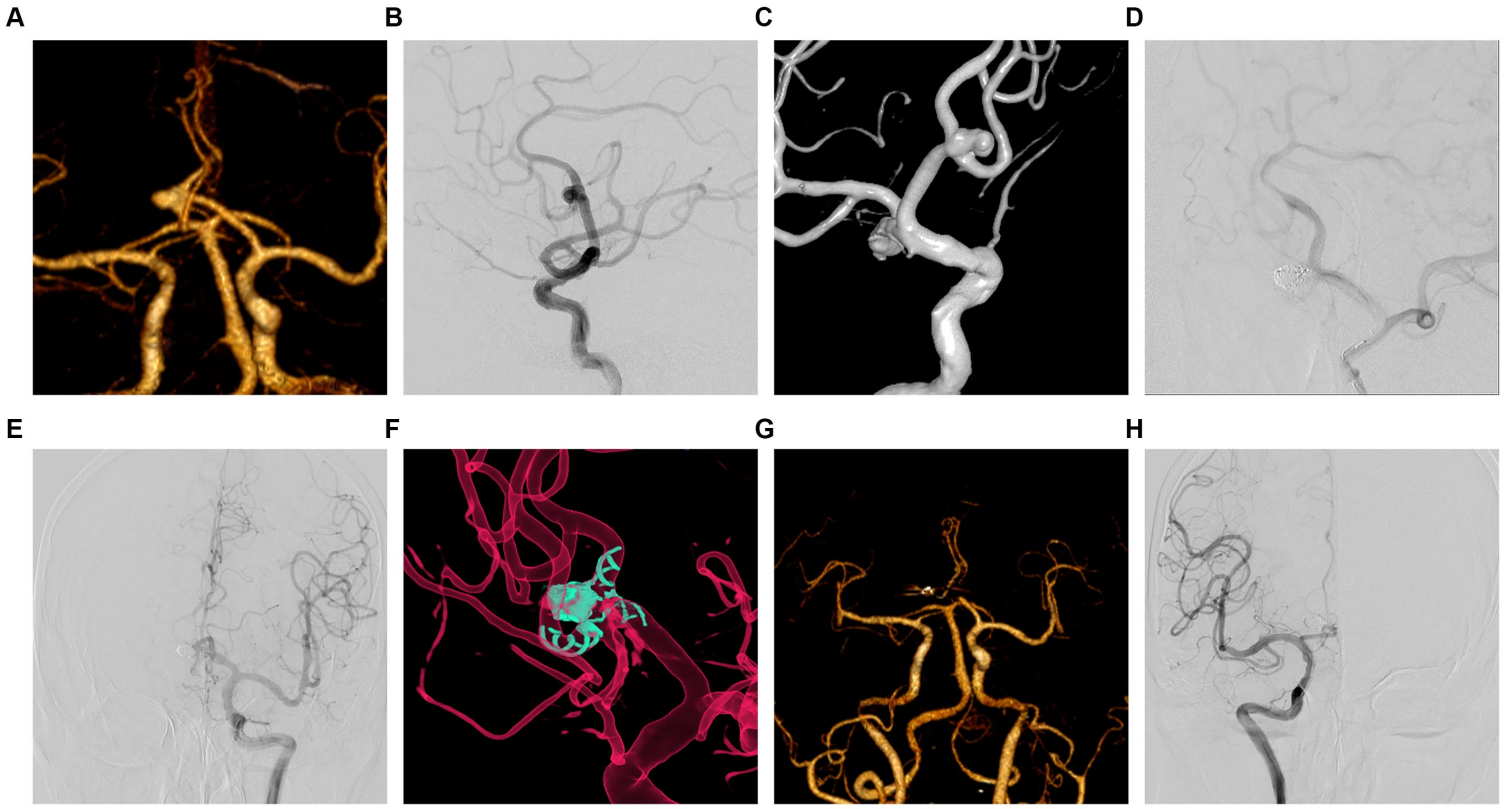
Intraoperative complications and postoperative follow-up period for embolization of unruptured AcomA patient. **(A)** A 67-year-old male, diagnosed with an AcomA aneurysm by CTA. **(B)** Cerebral angiography in the lateral position clearly showed an aneurysm in the AcomA. **(C)** 3D reconstruction clearly showed that the aneurysm in the AcomA involved both anterior cerebral arteries, and Y-configuration stent-assisted embolization was proposed for the treatment. **(D)** The right ACA was not visualized by anterior and lateral position angiography during the procedure. **(E)** Immediate administration of tirofiban was conducted through a guiding catheter and microcatheter, with a gradual sequential injection totaling 23 mL over 3 min. Simultaneously, a peripheral intravenous infusion was maintained at a dosage of 0.1 μg/kg/min. Following the completion of the injection, a subsequent lateral angiography demonstrated successful reperfusion of the anterior cerebral artery. **(F)** Bilateral ACA was well illuminated by 3D reconstruction in immediate postoperative angiography. **(G)** The bilateral ACA was well-luminated by CTA one week after surgery. **(H)** The right ACA was well-illuminated by cerebral angiography at 10 months after surgery.

**Figure 3:**
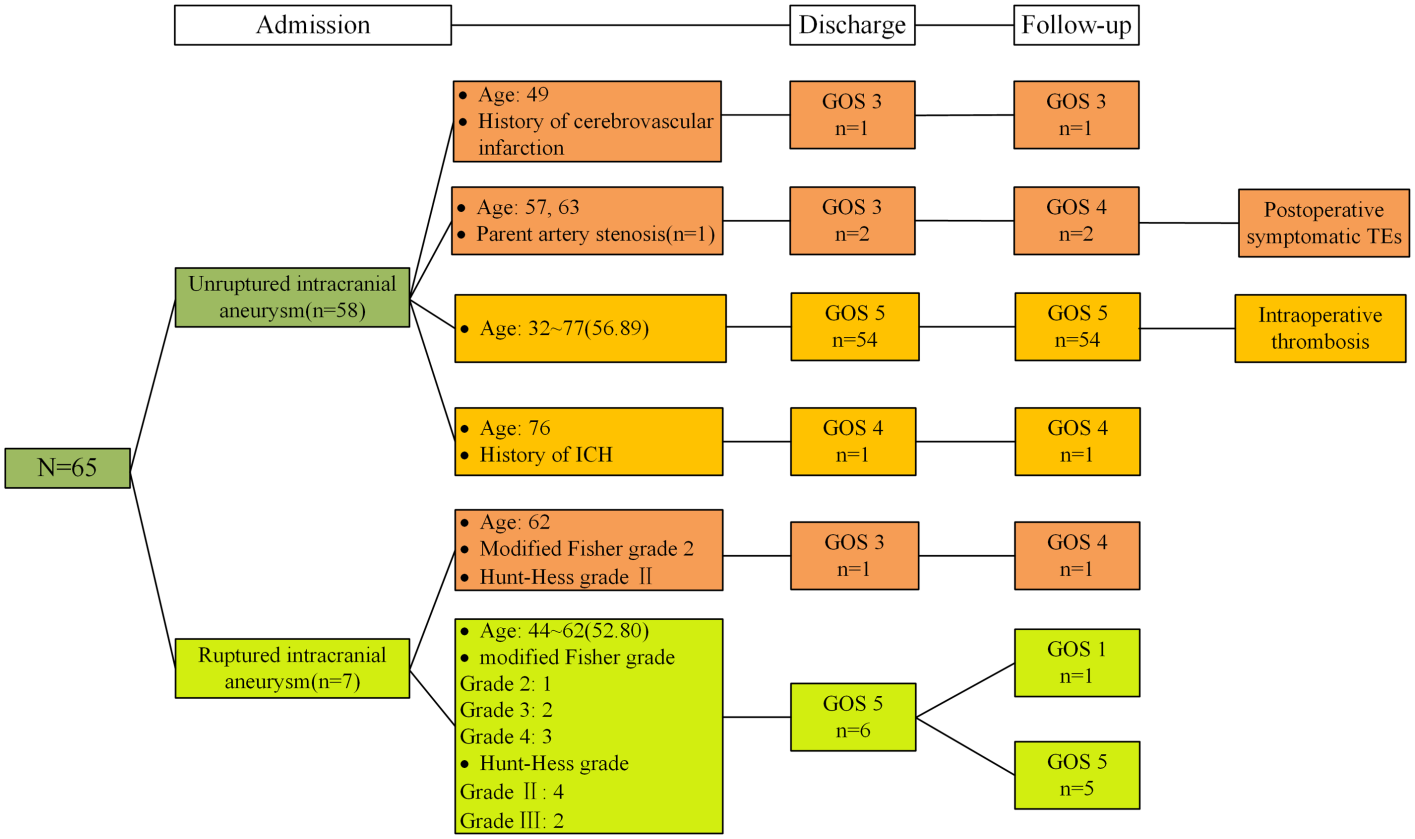
Admission status and clinical follow-up of all patients. ICH, intracerebral hemorrhage.

Among patients who underwent dual stent-assisted embolization with LEO stents of wide-necked intracranial bifurcation aneurysms, all 130 LEO stents were successfully implanted, and the stents were fully opened and well adhere to the artery, with no poor stent opening or kinking; moreover, none of the stents showed in-stent stenosis or coil displacement. The procedure times ranged from 1.3 to 6.22 h, with a mean procedure time of 3.00 ± 1.11 h. Immediately after the procedure, the mRR classification was assessed. We found mRRC I in 60 patients (92.3%), mRRC II in 3 patients (4.6%), and mRRC III b in 2 patients (3.1%) (Table 2). A total of 3 (4.6%) patients developed perioperative TEs; 1 patient developed intraoperative TEs, and the blood vessels were recanalized after a total of 20 mL of tirofiban was injected several times through the guiding catheter and the microcatheter; at the same time, tirofiban was pumped intravenously at a dosage of 0.1 μg/kg/min. The patient did not exhibit any obvious clinical symptoms after surgery, and the postoperative CTA were normal (Figure 2). Two patients developed symptomatic cerebral infarction within 3 days after surgery, examinations indicated that detachment of the thrombus might have led to distal occlusion of the aneurysm-carrying artery, and the patients’ symptoms relieved after active treatment, but limb mobility impairment continued before discharge. Upon discharge, both 2 patients presented with a GOS of 3 (Figure 3). Subsequent to the latest clinical follow-up assessment, both patients exhibited an amelioration in their GOS to 4 (Figure 3). Five patients developed perioperative bleeding, without serious bleeding events, including gingival bleeding, skin petechiae, and large petechiae at the puncture sites, and the symptoms improved after the dose of antiplatelet drugs was adjusted during the perioperative period. The patients’ GOS results were evaluated at the time of discharge; 60 patients had a GOS of 5, 1 had a GOS of 4, and 4 had a GOS of 3.

**Table 2:** Immediate post-operative and follow-up aneurysm occlusion rates.

Score	Results of mRR class	
	Immediate angiographic	Last follow-up DSA
mRRC I	60 (92.3%)	43 (95.6%)
mRRC II	3 (4.6%)	2 (4.4%)
mRRC III b	2 (3.1%)	0
Total	65	45

### 3.3 Follow-up results

**Figure 4:**
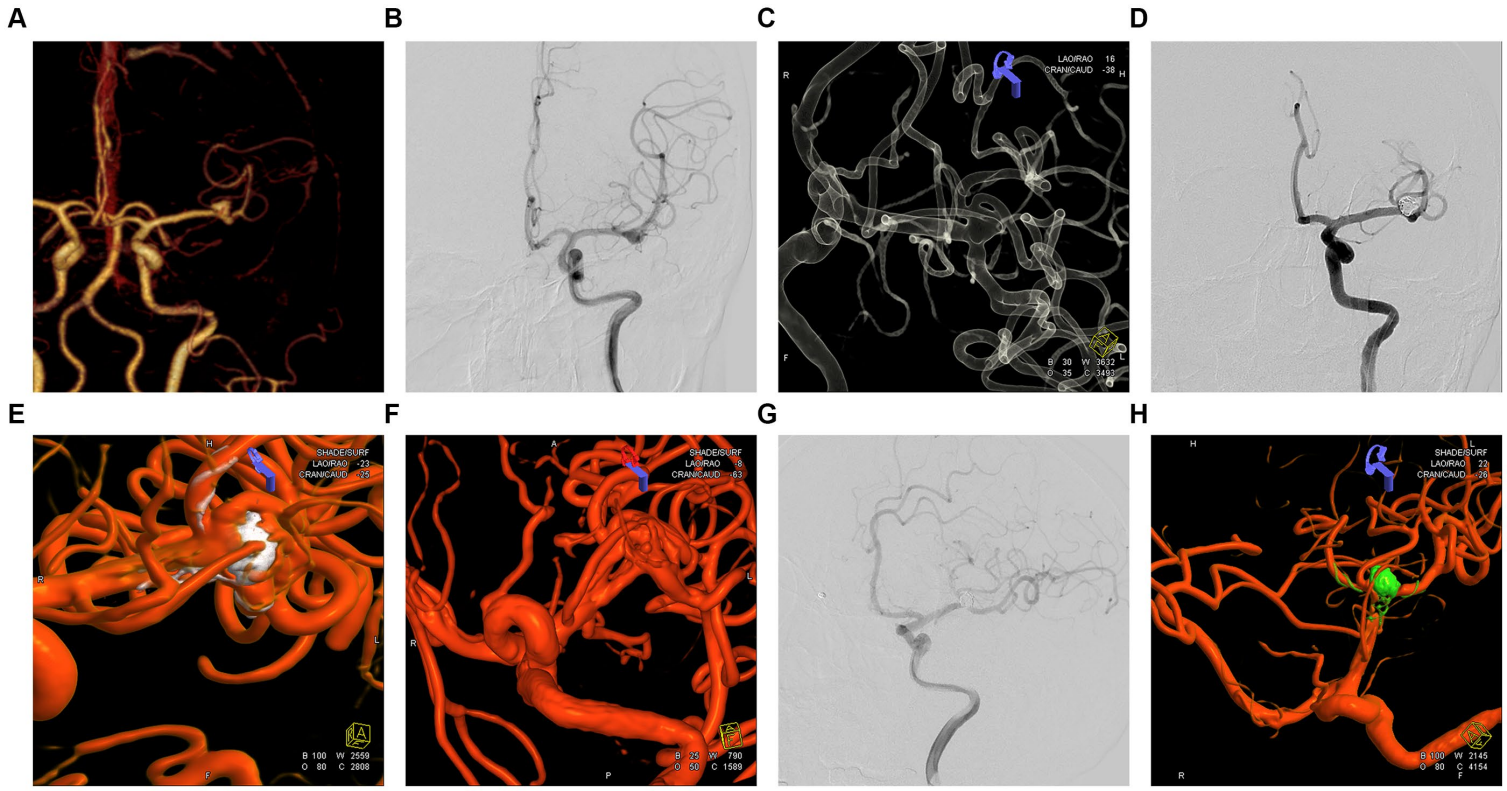
Embolization and imaging follow-up of an unruptured left middle cerebral artery (MCA) bifurcation aneurysm. **(A)** A 57-year-old woman was admitted for treatment of a left middle cerebral artery aneurysm diagnosed by CTA. **(B)** Orthostatic cerebral angiography revealed a wide-necked bifurcation aneurysm in the left MCA. **(C)** 3D reconstruction showing the left MCA aneurysm involving three middle cerebral artery branches, and Y-stent-assisted embolization was proposed for treating the aneurysm. **(D)** Postoperative 3D angiography showing embolization of the aneurysm in the left MCA bifurcation. **(E,F)** The 3D reconstruction reveals the embolization status of the aneurysm at the bifurcation of the left MCA. The embolization result is classified as mRRC III b, with partial opacification observed in the aneurysmal sac. **(G,H)** Postoperative orthopantomography and 3D reconstruction demonstrating improvement in the embolization of the aneurysm located in the branch of the left MCA at the seventh month after surgery, with the aneurysm completely and densely embolized to mRRC I.

We obtained the imaging follow-up data of 56 patients from 1 month to 2 years after surgery and recorded the cerebral angiography mRR classification at the latest follow-up visit. The duration of imaging follow-up ranged from 6 to 24 months, with a median follow-up time of 10 months. A total of 56 patients were imaged and followed up; 45 patients were followed up by DSA, and 11 patients were followed up by CTA or MRA. Postoperative DSA results revealed mRRC I in 43 patients (95%) and mRRC II in 2 patients (5%). Among these patients, 2 patients improved from aneurysm visualization to complete dense embolization (Figure 4), and 1 patient with mRRC II showed conversion to complete dense embolization (Table 2). There was no significant difference compared with that in the immediate postoperative period (*p* = 0.827); However, these cases showed improvement during follow-up. Eleven patients underwent only CTA or MRA follow-up without subsequent DSA reimaging. Neither CTA nor MRA revealed any apparent recurrence of intracranial bifurcation aneurysms. The patients underwent clinical follow-up assessments 1 to 24 months after surgery, and the last clinical follow-up assessment was performed 4 to 24 months after surgery, with a median follow-up of 7 months; 59 patients had a GOS of 5, 4 patients had a score of 4; and 1 patient had a score of 3 (Figure 3). Only 1 patient died of lung cancer during the follow-up period and had a GOS of 1; 3 patients had a GOS of 3 at the time of discharge from the hospital, and their GOS improved to 4 after all follow-up assessments were completed (Table 3). No significant difference was observed (*p* = 0.207), but patients with worse scores showed improvement during the clinical follow-up period.

**Table 3:** Discharge and follow-up GOS of the patients.

Scores	Results of GOS	
	Discharge	Last clinical follow-up
1	0	1 (1.5%)
2	0	0
3	4 (6.2%)	1 (1.5%)
4	1 (1.5%)	4 (6.2%)
5	60 (92.3%)	59 (90.8%)
Total	65	65

## 4 Discussion

Intracranial aneurysms are mainly treated by surgical microclamping and endovascular treatment, and surgical procedures are traumatic, have a long recovery time and are more difficult to be accepted by patients (14)^;^ therefore, endovascular treatment has gradually become the first choice. However, intracranial bifurcation aneurysms are complex and involve more branch arteries, especially wide-necked aneurysms; moreover, the use of traditional single stent-assisted embolization for bifurcation aneurysms is associated with a low rate of complete embolization and a high risk of recurrence in the follow-up period (15). In recent years, dual stent-assisted embolization is a new method for accessing wide-necked intracranial bifurcation aneurysms (3, 16). In particular, dual stent-assisted embolization with braided stents for the treatment of wide-necked intracranial bifurcation aneurysms has received much attention from peers in recent years because of its ability to repair the neck of the aneurysm effectively and its low recurrence rate. However, the simultaneous placement of two stents in the aneurysm-carrying artery increases the incidence of perioperative ischemic events, especially with the use of braided stent-assisted embolization, such as the use of LEO stents; therefore, this study examined the safety and efficacy of utilizing the dual stenting technique with LEO stents for assisting in the embolization of wide-necked intracranial bifurcation aneurysms. Additionally, we evaluated the perioperative effectiveness of dual antiplatelet drug therapy via oral administration and combined IA and IV intra-procedural infusion of tirofiban in preventing ischemic events and its influence on patients’ prognosis.

### 4.1 Safety and efficacy of dual stent-assisted embolization therapy

Dual stent-assisted embolization has emerged as an innovative treatment for wide-necked intracranial bifurcation aneurysms, addressing challenges associated with single stent-assisted embolization and potential recurrence due to branching arteries. Compared with open-loop stents, LEO stents have 95% remodelability; the greater metal coverage (18%) of LEO stents allows better blood flow and more effective repair of aneurysm necks than laser-engraved stents 9. Sixty-five patients with wide-necked intracranial bifurcation aneurysms were included in this study; two patients had ruptured aneurysms of the middle cerebral artery resulting in SAH, the two patients were graded as Grade II according to the Hunt–Hess classification (17, 18). This study, encompassing 65 patients, successfully employed dual-stent placement with a 100% success rate, showcasing no instances of poor stent opening or fracture. The stent configurations included 58 Y-shaped stents, 5 T-shaped stents, and 2 X-shaped stents. Immediate postoperative angiography revealed favorable outcomes in all cases, with 95.4% classified as mRRC I and II, and 3.6% as mRRC III (Table 2). No instances of spring coil displacement or stent-related stenosis were observed. Follow-up assessments after 6 to 24 months showed sustained positive outcomes, with all patients achieving mRRC I and II. Currently, numerous studies have demonstrated that web or Contour embolization is effective in treating bifurcation wide neck aneurysms. However, the overall efficacy of this embolization technique remains suboptimal. Some investigations have employed web-assisted embolization for these aneurysms, reporting a complete embolization rate of 90.5% at one year postoperatively (19). This rate is marginally lower than that observed in the present study. Moreover, there are significant challenges associated with the widespread implementation of insaccular flow modulating devices. However, the number of patients included in this study is relatively small and further exploration is needed. Several meta-analyses have examined the use of WEB or Contour devices in the endovascular treatment of wide-neck bifurcation aneurysms, with reported ischemic event incidences ranging from 4.1 to 8.53% (20–22). In our study, the incidence of ischemic events was 4.6%. However, it should be noted that the application of WEB and Contour devices is constrained by certain limitations, particularly in the treatment of specific types of aneurysms. Even patients with initially poor embolization results exhibited improvement, and no in-stent stenosis was detected. Notably, the use of LEO stents in a T-shaped configuration demonstrated a high success rate of approximately 95.8% in a similar study. Comparative analyses and meta-analyses, including a study of 721 patients, supported the safety and efficacy of dual stent-assisted coiling for complex intracranial aneurysms. Procedure-related complications were reported at 7.7%, with a technical success rate of 97.6% and an aneurysm complete occlusion rate of 88.4% (23). The type of stent and its successful placement need to be determined according to the morphology of the intracranial aneurysm and the experience of the operator; all dual stent techniques in this study were successfully implemented. No complications related to stent malapposition, stent recoil, or other surgery-related issues were observed. The current study exhibited a 100% follow-up success rate in achieving mRRC I and II, surpassing previous studies using carved stents and rivaling those using LEO stents in Y-shaped techniques. In a retrospective study of dual stent-assisted embolization for wide-necked intracranial bifurcation aneurysms, 73% of patients achieved immediate postoperative mRRC I and II, aligning with our results (23). Another study using Y-stenting with LEO stents reported an immediate postoperative mRRC I and II rate of 99.1%, surpassing other techniques (24). The immediate postoperative embolization effectiveness in this study outperformed the use of carved stents for dual stent-assisted embolization therapy. Similar to the postoperative angiography results of the LEO stent Y-shaped technique, but with better follow-up outcomes. This superiority is attributed to the enhanced metal coverage of LEO stents, providing improved blood flow guidance and aneurysm neck repair. A study on dual stent-assisted embolization using LEO stents for small artery aneurysms reported 60% immediate postoperative mRRC I and II, emphasizing the potential influence of aneurysm-carrying artery diameter on outcomes (25). In another retrospective study utilizing X and Y stent-assisted coiling techniques for treating intracranial aneurysms, the immediate postoperative embolization effectiveness in mRRC I and II was approximately 64.8%. However, during the follow-up period, the embolization effectiveness in mRRC I and II reached 92.9% (3). Notably, both the immediate and follow-up embolization effectiveness in this study were comparatively lower than those observed in our research. This variance might be attributed to the utilization of LEO stent assistance in our study as opposed to the relatively limited use of braided stents like LEO in the aforementioned study. The rate of near-complete occlusion of wide-necked intracranial bifurcation aneurysms during the follow-up period in our center was 100%. The employment of LEO stents in dual-stent-assisted embolization therapy for wide-necked intracranial bifurcation aneurysms has exhibited a notable efficacy, characterized by a high success rate. The utilization of diverse stent configuration techniques has consistently yielded commendable embolization outcomes, characterized by robust neck reconstruction and minimal recurrence rates. This underscores the efficacy of this approach as a compelling and efficient method for the treatment of wide-necked intracranial bifurcation aneurysms.

### 4.2 Prevention of perioperative TEs

Braided stents, known for facilitating aneurysm neck repair and reducing postoperative recurrence risks, are accompanied by thrombogenicity concerns during stent-assisted embolization of intracranial aneurysms, especially when deploying two stents in an aneurysm-carrying artery (26). To address the elevated risk of thrombosis, rational antiplatelet therapy becomes imperative. Tirofiban, a nonpeptide platelet surface glycoprotein IIb/IIIa (GP IIb/IIIa) antagonist, presents multiple thrombolytic effects, including inhibiting platelet aggregation, blocking the release of local thrombolytic inhibitors, and attenuating clot structure. Furthermore, it stimulates endothelial cell migration and proliferation, fostering vascular endothelium repair post-stent implantation. Remarkably, substantial platelet inhibition is achieved within 5 to 10 min of tirofiban administration (27). Our study involving 65 patients with intracranial aneurysms treated with dual stent-assisted embolization using LEO stents, perioperative TEs were successfully prevented by combining oral dual antiplatelet drugs with combined IA and IV intra procedural infusion of tirofiban. The incidence rate of perioperative thromboembolic events was approximately 4.6%, with no serious perioperative bleeding events observed. A follow-up assessment showed no significant difference in the GOS compared with the discharge score (*p* = 0.207), but patients with initially worse GOS exhibited improvement. Comparatively, a study focusing on dual-stent-assisted coiling for intracranial aneurysms reported a higher incidence of perioperative TEs (7.7%) with the administration of oral dual antiplatelet therapy (23). Notably, there were no surgery-related bleeding complications. To enhance perioperative TEs prevention, the concurrent use of tirofiban via arterial and venous routes demonstrated superior efficacy, highlighting its heightened antiplatelet potency and the quicker recovery of platelet function upon discontinuation. Importantly, this approach did not report any severe surgery-related bleeding complications. In another study addressing Y-stent-assisted coiling for acutely ruptured wide-necked intracranial aneurysms, a significant reduction in the incidence of perioperative TEs (13.3%) was achieved by employing a rapid intravenous bolus of tirofiban at a high dose (8ug/kg) alongside a sustained low-dose (0.1ug/kg/min) continuous infusion over 4 to 6 h (28). The higher rate of perioperative TEs in this study was attributed to inadequate preoperative antiplatelet therapy for ruptured aneurysms and the use of intravenous tirofiban alone, resulting in a higher incidence compared to the mentioned study. A retrospective meta-analysis of 38 studies reported an incidence of perioperative hemorrhagic events ranging from 1.3 to 3.8% in patients undergoing dual stent-assisted embolization therapy (23). However, in the current study, no severe perioperative bleeding events were recorded, indicating a lower incidence compared to the referenced study. This discrepancy in bleeding rates was attributed to the administration of tirofiban, facilitating a prompt restoration of platelet function upon discontinuation. A recent meta-analysis investigating the utilization of flow diversion-assisted embolization for intracranial bifurcation aneurysms reported TEs and HEs incidences of approximately 16 and 4%, respectively (29). In the present study, the incidence of TEs was notably lower compared to the meta-analytic findings, while the incidence of HEs was marginally higher; however, it is critical to note that no severe bleeding events were observed. These results underscore the enhanced safety profile and efficacy of tirofiban in our cohort, suggesting its superior reliability in clinical application for such cases. Despite more studies focusing on perioperative TEs in patients receiving dual stent-assisted embolization for wide-necked intracranial bifurcation aneurysms, fewer studies explore the perioperative use of tirofiban following oral dual antiplatelet drugs, particularly in an arterial–venous combination (3). In the center conducting this study, the incidence of perioperative TEs due to combined IA and IV intra procedural infusion of tirofiban antiplatelet therapy during dual stent-assisted embolization for intracranial aneurysms was only 4.6%. The use of intra-arterial infusion of tirofiban after acute intraoperative thrombosis also proved significantly effective without increasing the risk of bleeding (30, 31). In conclusion, the comprehensive approach of integrating oral dual antiplatelet drugs with combined IA and IV intra-procedural infusion of tirofiban shows promising outcomes in mitigating perioperative TEs during dual stent-assisted embolization for intracranial aneurysms. This method, supported by the administration of tirofiban, not only ensures efficient antiplatelet activity but also contributes to a faster recovery of platelet function without imposing a significant bleeding risk. Ongoing research should further explore the application of tirofiban in perioperative strategies for wide-necked intracranial bifurcation aneurysms to optimize outcomes and minimize complications (23, 32).

## 5 Limitations

There are several limitations to this study. Firstly, it is a single-center retrospective study. Secondly, the absence of a control group introduces a notable limitation, which we intend to address in future studies by incorporating a relevant control group. Lastly, this study did not routinely monitor postoperative thromboelastogram (TEG) and inhibition of platelet aggregation. Additionally, DWI scans were not routinely performed on all postoperative patients, which may result in a high incidence of asymptomatic ischemic events.

## 6 Conclusion

Our study suggests that dual stent-assisted embolization using LEO stents is a safe and effective treatment for wide-necked intracranial bifurcation aneurysms. Perioperative TEs can be effectively prevented and managed through the implementation of perioperative oral dual antiplatelet therapy in conjunction with combined IA and IV intra-procedural infusion of tirofiban. Furthermore, no secondary intracranial hemorrhagic complications were observed.

## Data availability statement

The raw data supporting the conclusions of this article will be made available by the authors, without undue reservation.

## Ethics statement

The studies involving humans were approved by the Medical Ethics Committee of the First Affiliated Hospital of Army Medical University. The studies were conducted in accordance with the local legislation and institutional requirements. Written informed consent for participation was not required from the participants or the participants' legal guardians/next of kin in accordance with the national legislation and institutional requirements.

## Author contributions

KW: Writing – original draft, Data curation, Investigation, Methodology. ZL: Conceptualization, Methodology, Writing – review & editing. ZZ: Data curation, Writing – review & editing, Investigation. JT: Data curation, Writing – review & editing, Investigation. CY: Data curation, Writing – review & editing, Investigation. BY: Investigation, Writing – review & editing, Resources, Supervision. GZ: Supervision, Writing – review & editing, Funding acquisition, Resources. HM: Funding acquisition, Supervision, Writing – review & editing, Conceptualization, Methodology.
